# Supplementary material for: An MRI Radiomics Approach to Predict the Hypercoagulable Status of Gliomas
Source: Cancers (Basel). 2024 Mar 26;16(7):1289. doi: 10.3390/cancers16071289 (PMC11010849; doi:10.3390/cancers16071289)
Supplement: Supplementary file 1 [file cancers-16-01289-s001.zip › cancers-2884180-supplementary/Supplementary Figures and Tables.pdf]

Supplementary Figures and Tables

A Magnetic Resonance Imaging Radiomics Approach to Predicting the Hypercoagulable Status of Gliomas

Zuzana SAIDAK *et al.*

Figure S1 : Basic clinical characteristics of TCGA and REMBRANDT cohorts

Figure S2 : Histograms of *F3* expression in TCGA and REMBRANDT.

Figure S3 : Confusion matrices for LGG/GBM in TCGA and REMBRANDT cohorts.

Figure S4: ROC analysis of the Radscore for LGG and GBM in TCGA.

Figure S5 : Tumor aneuploidy and chromosomal instability scores in LGG and GBM in TCGA

Figure S6 : CIBERSORTx analysis of immune cell relative fractions and absolute scores according to Radscore in TCGA

Table S1 : Radscore model characteristics

Table S2 : Median survival and Hazard ratios according to the Radscore

Table S3 : GSEA leading edge analysis for the Hallmark « COAGULATION » in gliomas stratified according to Radscore

Table S4 : Absolute immune infiltration levels in gliomas stratified according to the Radscore in TCGA

Table S5 : Pearson correlations between the Radscore and Immune infiltrate (CIBERSORTx absolute) in TCGA LGG/GBM.

Figure S1 : Basic clinical characteristics of TCGA and REMBRANDT cohorts

The proportions of basic clinical data, including age, sex, histological subtype and grade were compared between TCGA (n=136) and REMBRANDT (n=39) cohorts using Chi2.

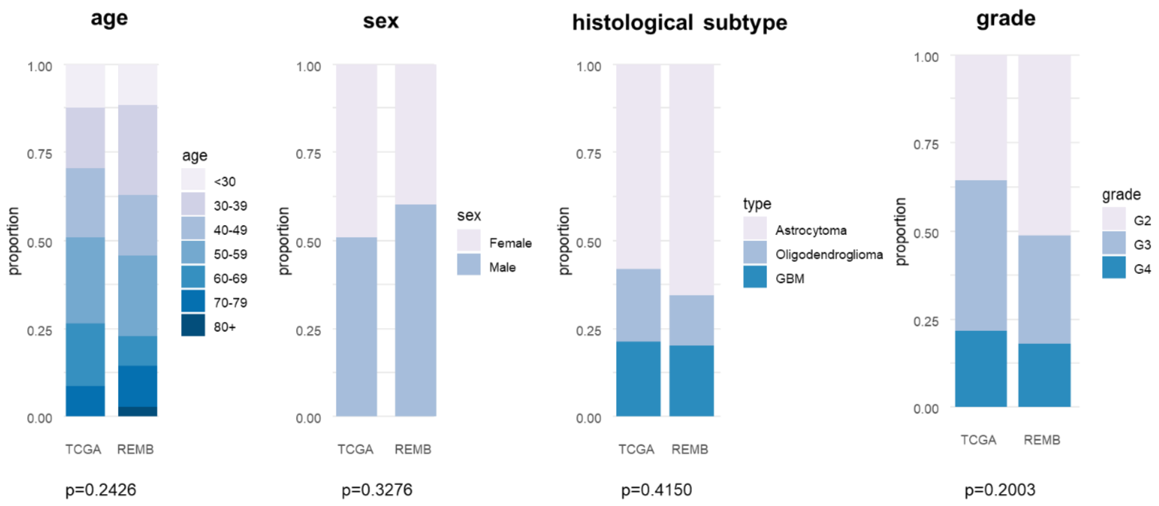

Figure S2: Histograms of *F3* expression in TCGA and REMBRANDT.

Histograms showing the distribution of *F3* (TF) expression in the TCGA (n=136) and REMBRANDT (n=39) cohorts. The top 20% highest *F3* expressing tumors are indicated in red.

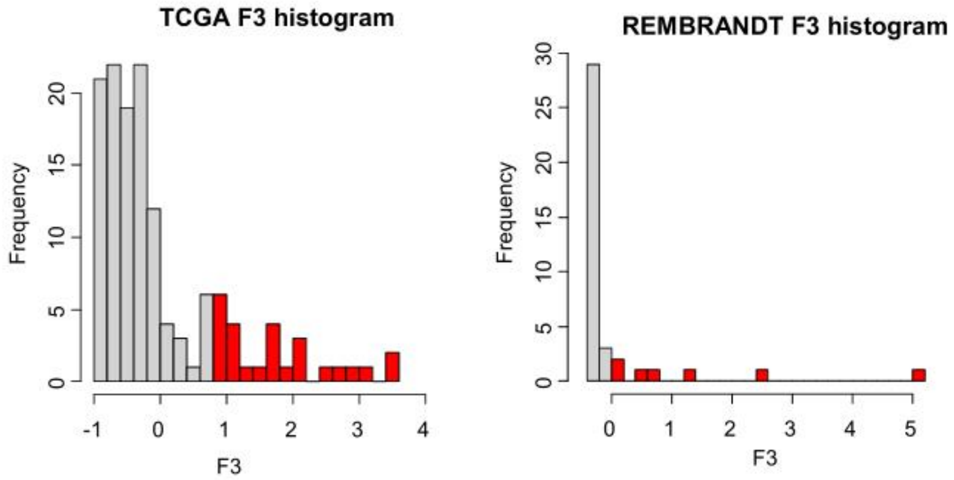

Figure S3 : Confusion matrices for LGG/GBM in TCGA and REMBRANDT cohorts.

Confusion matrix for TCGA		REAL	
		**1**	**0**
**PREDICTED**	**1**	24	26
	**0**	3	83

Confusion matrix for REMBRANDT		REAL	
		**1**	**0**
**PREDICTED**	**1**	6	9
	**0**	1	23

Figure S4: ROC analysis of the Radscore for LGG and GBM in TCGA.

Performance analysis (ROC) of the Radscore in identifying the top 20% F3^{high} tumors in the TCGA-LGG and TCGA-GBM cohorts. The shaded area indicates the IC95.

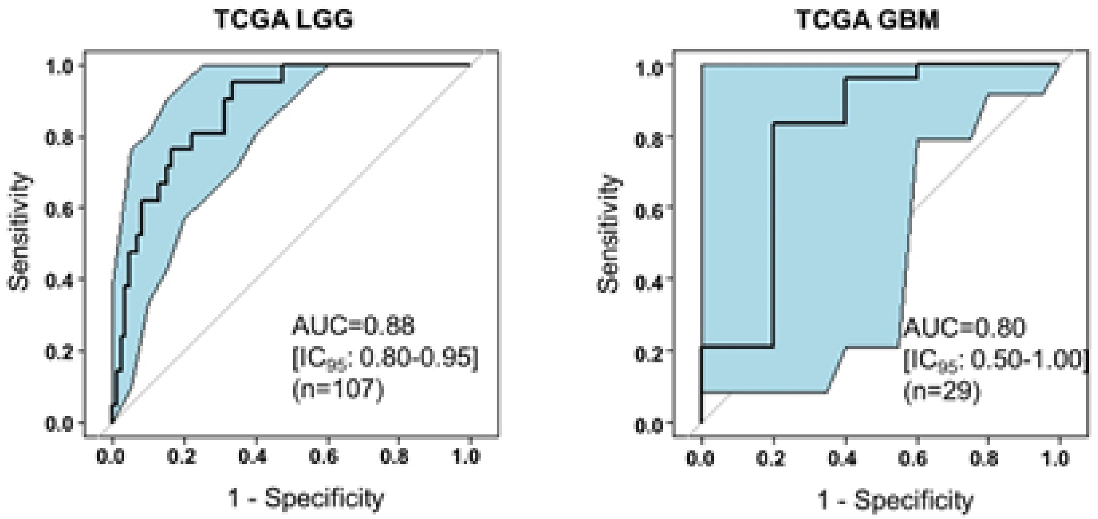

Figure S5: Tumor aneuploidy and chromosomal instability scores in LGG and GBM in TCGA.

Comparison of the aneuploidy and CIN70 scores in TCGA-LGG and TCGA-GBM stratified according to the Radscore (Wilcoxon-Mann-Whitney).

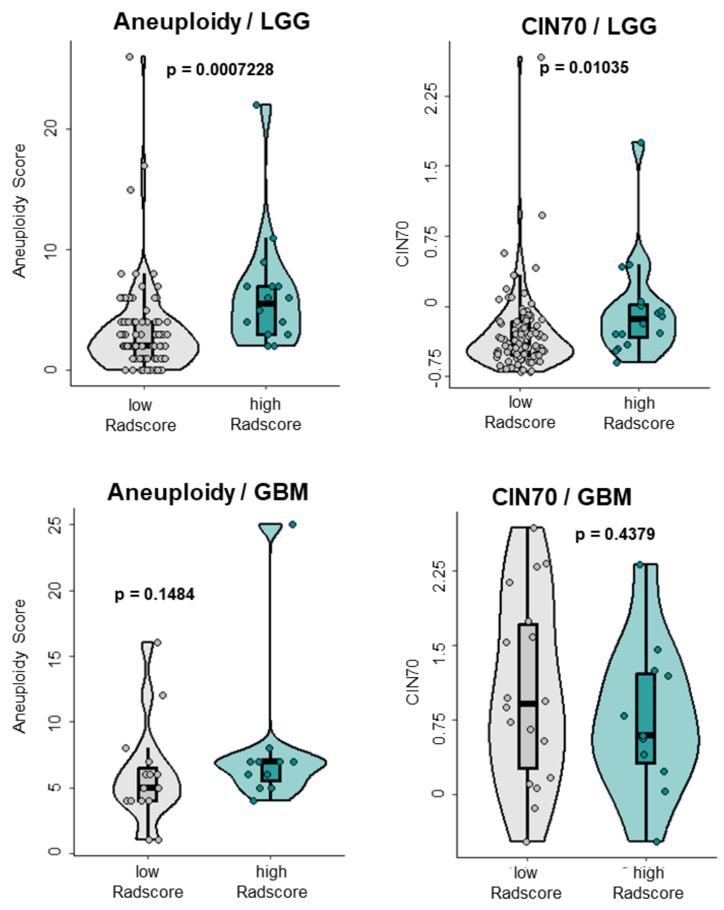

Figure S6 : CIBERSORTx analysis of immune cell relative fractions and absolute scores according to Radscore in TCGA.

Relative fractions of 22 immune cell types (left) or absolute scores (right), determined using CIBERSORTx, were compared in the TCGA cohort according to the Radscore (Low *vs* High).

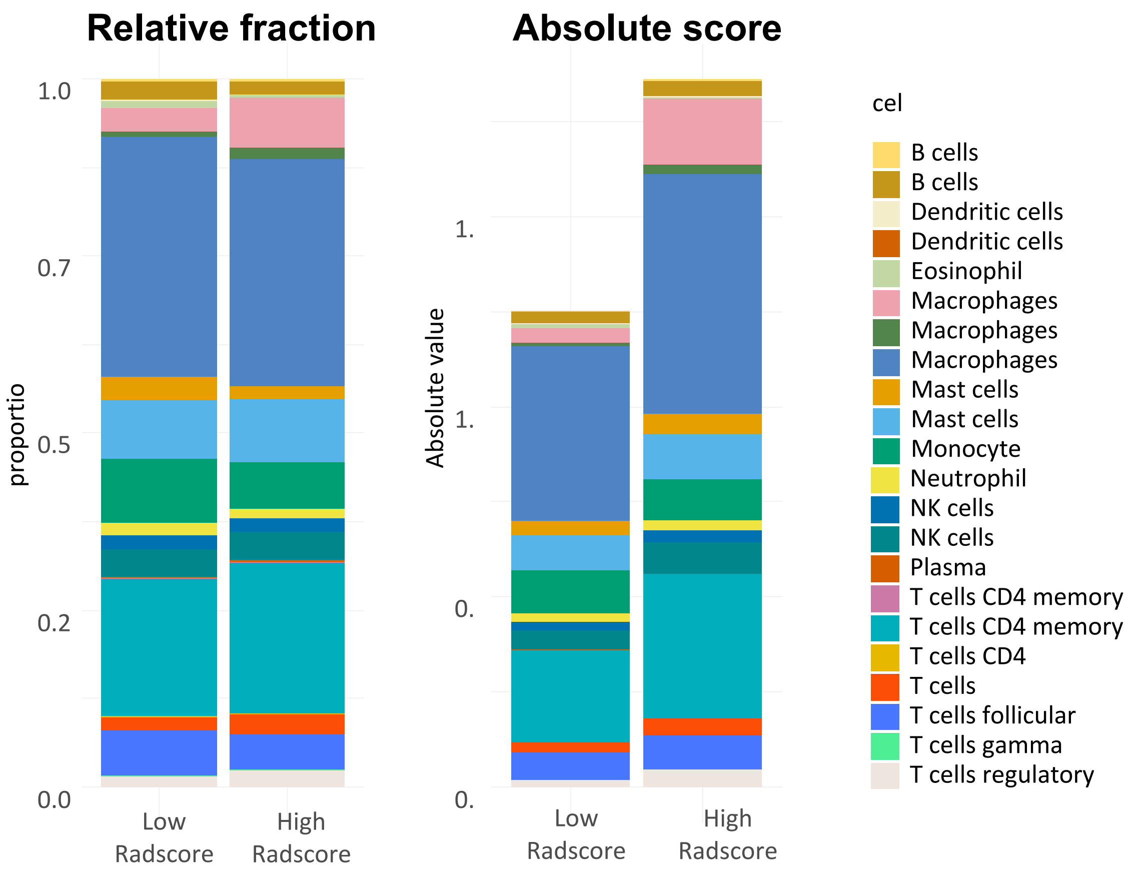

Table S1: Radscore model characteristics

Table showing the values of the coefficients for each of the 7 features retained in the Radscore model

Feature	coefficient
glcm_Correlation	1.44
glcm_JointEnergy	1.44
glcm_InverseVariance	-1.11
gldm_LargeDependenceLowGrayLevelEmphasis	0.85
glrlm_RunVariance	-2.51
shape_Flatness	-0.85
shape_MeshVolume	-3.85

Table S2 : Median survival and Hazard ratios according to the Radscore

Table showing the median overall survival (OS) and disease-free survival (DFS) in TCGA and REMBRANDT patients according to the Radscore group, as well as the Hazard ratios for Radscore^{high} tumors.

	Median survival (months)		
	Radscore^{high}	Radscore^{low}	Hazard ratio, CI_{95}
TCGA/LGG OS	33.94	94.45	3.96 [1.58-9.94]
TCGA/LGG DFS	19.19	53.52	5.11 [1.90-13.76]
TCGA/GBM OS	10.61	14.72	3.32 [0.96-11.43]
TCGA/GBM DFS	3.21	10.22	1.45 [0.42-5.00]
REMBRANDT/LGG OS	13.00	56.60	2.75 [0.76-9.90]
REMBRANDT/GBM OS	8.00	22.80	3.09 [0.51-18.75]

Table S3: GSEA leading edge analysis for the Hallmark « COAGULATION » in gliomas stratified according to Radscore

Gene	Signal
ANXA1	0.5665
F3	0.5071
GNG12	0.4437
SERPING1	0.4376
BMP1	0.4267
CPQ	0.3965
ACOX2	0.3632
MMP14	0.3523
CFI	0.3441
PROS1	0.3404
TMPRSS6	0.3383
WDR1	0.3364
S100A13	0.3339
RGN	0.3259
C1R	0.3231
C1S	0.3151
ANG	0.3127
CD9	0.3006
ADAM9	0.2945
CSRP1	0.2902
P2RY1	0.2865
GNB2	0.2852
ITGA2	0.2846
PLAU	0.2843
PLAT	0.2799
DUSP6	0.2780
DUSP14	0.2752
CTSK	0.2676
ARF4	0.2643
F2RL2	0.2536
MMP2	0.2524
PROC	0.2498
C3	0.2411
SERPINA1	0.2405
TIMP1	0.2319
LAMP2	0.2246
SERPINC1	0.2216
DPP4	0.2190
COMP	0.2119
CRIP2	0.2083
PECAM1	0.2075

**APOC2**	**0.2066**
**MMP7**	**0.2027**
**MMP9**	**0.2022**
**CLU**	**0.2000**
**CFD**	**0.1978**
**F9**	**0.1977**
**CTSB**	**0.1958**
**FGA**	**0.1880**
**ITGB3**	**0.1873**
**CFB**	**0.1815**
**APOC3**	**0.1797**
**CAPN2**	**0.1782**
**APOC1**	**0.1721**
**PEF1**	**0.1706**
**MMP11**	**0.1659**
**OLR1**	**0.1653**
**FN1**	**0.1587**
**FGG**	**0.1550**
**MASP2**	**0.1550**
**FURIN**	**0.1495**
**APOA1**	**0.1485**
**HRG**	**0.1475**
**KLKB1**	**0.1444**
**LRP1**	**0.1425**
**C8B**	**0.1422**
**C1QA**	**0.1400**
**C8A**	**0.1378**
**PRSS23**	**0.1370**
**PLG**	**0.1328**
**F8**	**0.1318**
**SERPINE1**	**0.1294**
**MMP3**	**0.1252**
**F2**	**0.1251**
**F11**	**0.1233**
**A2M**	**0.1216**

Note that the genes are ranked according to their contribution to the Enrichment score (ES) in the GSEA analysis of TCGA-LGG/GBM stratified according to Radscore. Genes that were also identified by GSEA in the REMBRANDT cohort are highlighted in grey.

Table S4 : Absolute immune infiltration levels in gliomas stratified according to the Radscore in TCGA

	**Low Radscore (n=109)**	**High Radscore (n=27)**	**p (FDR)**
B cells naive	0.0302 [0.0208-0.0397]	0.0384 [0.0033-0.0734]	1
B cells memory	0.0042 [0.0028-0.0055]	0.0062 [0.0015-0.0109]	1
Plasma cells	0.0015 [0.0009-0.0021]	0.0011 [0.0002-0.0021]	1
T cells CD8	0.0256 [0.0172-0.0340]	0.0486 [0.0255-0.0717]	0.5632
T cells CD4 naive	0.0012 [0-0.0024]	0 [0-0]	1
T cells CD4 memory resting	0.2415 [0.2063-0.2766]	0.3781 [0.2675-0.4887]	0.0572
T cells CD4 memory activated	0.0002 [0-0.0005]	0.0002 [0-0.0005]	1
T cells follicular helper	0.0721 [0.0541-0.0902]	0.0874 [0.0590-0.1159]	1
T cells regulatory (Tregs)	0.0191 [0.0143-0.0238]	0.0457 [0.0248-0.0666]	0.0044
T cells gamma delta	0.0003 [0-0.0008]	0.0 [0-0]	1
NK cells resting	0.0500 [0.0382-0.0617]	0.0823 [0.0388-0.1259]	0.9482
NK cells activated	0.0241 [0.0180-0.0302]	0.0315 [0.0116-0.0513]	1
Monocytes	0.1142 [0.0958-0.1327]	0.1112 [0.0785-0.1439]	1
Macrophages M0	0.0382 [0.0245-0.0520]	0.1709 [0.0481-0.2937]	0.0017
Macrophages M1	0.0098 [0.0062-0.0135]	0.0260 [0.0083-0.0437]	0.1012
Macrophages M2	0.4563 [0.3810-0.5317]	0.6312 [0.4146-0.8477]	1
Dendritic cells resting	0.0002 [0-0.0005]	0.0003 [0-0.0007]	1
Dendritic cells activated	0.0023 [0.0009-0.0036]	0.0036 [0-0.0070]	1
Mast cells resting	0.0907 [0.0728-0.1087]	0.1178 [0.0656-0.1700]	1
Mast cells activated	0.0418 [0.0230-0.0605]	0.0522 [0-0.1046]	1
Eosinophils	0.0090 [0.0057-0.0124]	0.0049 [0.0020-0.0078]	1
Neutrophils	0.0214 [0.0160-0.0267]	0.0259 [0.0114-0.0403]	1

Note that the values analyzed here are absolute scores determined with CIBERSORTx.

Values are given as average [CI95], with *p* values obtained with Student's t test after FDR correction.

Cell types with p<0.05 (FDR) are highlighted in grey.

Table S5. Pearson correlations between the Radscore and Immune infiltrate (CIBERSORTx absolute) in TCGA LGG/GBM

	**Pearson R**	**p**
B cells naive	-0.05	0.5310
B cells memory	0.22	0.0096
Plasma cells	-0.01	0.8774
T cells CD8	0.20	0.0225
T cells CD4 naive	-0.04	0.6382
T cells CD4 memory resting	0.40	1.43e-06
T cells CD4 memory activated	0.06	0.4827
T cells follicular helper	0.09	0.2931
T cells regulatory (Tregs)	0.37	7.95e-06
T cells gamma delta	-0.04	0.6148
NK cells resting	0.23	0.0070
NK cells activated	0.07	0.4102
Monocytes	-0.04	0.6405
Macrophages M0	0.32	0.0001
Macrophages M1	0.16	0.0695
Macrophages M2	0.15	0.0905
Dendritic cells resting	0.02	0.8535
Dendritic cells activated	0.07	0.4229
Mast cells resting	0.21	0.0127
Mast cells activated	0.01	0.8984
Eosinophils	-0.10	0.2317
Neutrophils	0.14	0.1157

Cell types with p<0.05 are highlighted in grey.
