# Supplementary material for: An MRI Radiomics Approach to Predict the Hypercoagulable Status of Gliomas
Source: Cancers (Basel). 2024 Mar 26;16(7):1289. doi: 10.3390/cancers16071289 (PMC11010849; doi:10.3390/cancers16071289)
Supplement: Supplementary file 1 [file cancers-16-01289-s001.zip › cancers-2884180-supplementary/Supplementary Materials and Methods.pdf]

Supplementary Materials and Methods

A Magnetic Resonance Imaging Radiomics Approach to Predicting the Hypercoagulable Status of Gliomas

Zuzana SAIDAK *et al.*

Patient information and data availability

The Cancer Genome Atlas (TCGA) LGG and GBM cohorts consist of 516 and 606 patients, respectively. Newly diagnosed LGG and GBM were classified and graded according to histological criteria (oligodendroglioma, oligoastrocytoma, astrocytoma and glioblastoma; grades II to IV), as reported in detail in Ceccarelli *et al.* [36]. For each tumor sample analyzed in our study, we retrieved data obtained from RNA sequencing, DNA sequencing, copy-number analysis (single-nucleotide polymorphism arrays) from cBioPortal (https://www.cbioportal.org/) (1st November 2023). The REMBRANDT cohort is a collection of genomic data for brain tumors that was launched as a joint initiative by the NIH's National Cancer Institute (NCI) and National Institute of Neurological Disorders and Stroke (NINDS). Brain tumor samples were collected from 14 contributing institutions between 2004 and 2006. Tumor transcriptomic data are accessible via the Gene Expression Omnibus (GEO) repository (GSE108476).

F3 expression analysis

RNA sequencing data from TCGA were retrieved using CBioportal and are normalized using the RSEM method (RNA-seq by expectation maximization). RNA expression levels from REMBRANDT were retrieved from GEO (GSE108476). Importantly, RNA expression was analyzed by microarray (Affymetrix U1332) in this cohort. When multiple rows were available for the same gene, the maximal value was retained. Microarray analysis indeed relies on the use of a small number of probes for each gene. Conflicting information can be obtained, for example if a specific splicing variant is only detected with one probe. We chose to retain in each case the maximal expression value, in order to analyse the most expressed mRNA species. To allow for inter-cohort comparison, *F3* gene expression values were normalised to Z scores within each cohort.

Radiomics features, handling of missing information

Briefly, we downloaded radiomics features that had been extracted from pre-operative MRI images of LGG/GBM patients in previous studies by Bakas et al. [38] and Sayah et al. [39]. The radiomics features (n=120) had been obtained after applying the same pipeline to the two cohorts, including automated

volumetric segmentation of MRIs (GLISTERboost), used to identify the tumor necrotic core, edema, Non-Enhancing Tumor (NET) and Enhancing Tumor (ET), Grey Matter (GM), white matter (WM) and Cerebrospinal Fluid (CSF), followed by an independent verification of label segmentation by certified neuroradiologists [38,39]. As reported in detail in the corresponding research papers, 120 features were extracted volumetrically by the same team and consisted of i) intensity, ii) volumetric, iii) morphologic, iv) histogram-based and v) textural parameters, including features based on wavelets, Gray Level Co-occurrence Matrix (GLCM*),* Gray Level Dependence Matrix (GLDM), Gray-Level Run-Length Matrix (GLRLM), Gray-Level Size Zone Matrix (GLSZM), and Neighborhood Gray-Tone Difference Matrix (NGTDM) [38,39]. Radiomics data were available for 243 LGG/GBM patients in the TCGA cohort and for 64 patients in REMBRANDT. Missing radiomics values (0.16% of values) were imputed using the median. Radiomics features were normalised to Z scores. In the end, complete radiomics and transcriptomic data were available for n=136 tumors in TCGA and n=39 tumors in REMBRANDT.

Radscore construction

The radiomics score (Radscore) is the sum of the product of each feature with its corresponding coefficient (Radscore = $\Sigma\beta_i * x_i$ + intercept), where x_i and β_i are the retained features and corresponding coefficients. The Radscore was calculated for each tumor before analysis. Upon application to LGG/GBM of TCGA, the Radscore range was : -20.69 to 14.55.

Evaluation of model performance

The Receiver Operating Characteristic (ROC) curve was used to assess the predictive power of the Radscore, using the Area Under the Curve (AUC) as a readout. The model performance was also assessed using standard methods: sensitivity = TP/(TP+FN), specificity = TN/(FP+TN), positive predictive value = TP/(TP+FP), negative predictive value = TN/(TN+FN) and Accuracy = (TP+TN)/(TP+FP+TN+FN).

Tumor genomic data, aneuploidy score, CIN70 score

IDH mutational status and EGFR copy number alterations were retrieved from cBioportal (https://www.cbioportal.org/) on the 1st of November 2023. The aneuploidy scores were retrieved from Thorsson *et al.* and reflect the total sum of amplified or deleted chromosomal arms [44] (aneuploidy score range for TCGA LGG/GBM: 0 to 26). The CIN70 (Chromosomal Instability) score is a 70-gene mRNA expression signature that has been reported by Carter *et al.* [42] (CIN70 range for TCGA LGG/GBM : -0.71 to 2.70).

List of R packages used

**Training and feature selection**	**nestedcv** version 0.7.4 package (with **glmnet** version 4.1-8 and **caret** version 6.0-94), alpha=1 (LASSO), "binomial" family	In order to identify the most important features linked to F3^{high}, we performed a repeated ten-fold CV process, repeated 10 times. Lambda.min and Lambda.1se were determined in order to select the optimal number of parameters to retain (stability).
**Creation of a logistic regression model**	R function **glm**, "binomial" family	A 7-feature model was created (Radscore)
**ROC analysis / model performance**	**OptimalCutpoints** version 1.1-5 package, methods "MaxProdSpSe", **epiR 2.0.68** package and **pROC** package 1.18.5	optimal ROC curve cutoff, maximizing the product sensitivity x specificity
**Survival analysis**	R packages **survival** 3.5-8, **survminer** 0.4.9 , **lattice** 0.22-5 log rank test for comparisons	To compare overall survival (OS) and disease free survival (DFS) according to Radscore

**Graphical presentation**	**ggplot2** version 3.4.4	Violin plots, survival analyses
**Immune score analysis**	R package **tidyestimate**	To evaluate tumor purity using the ESTIMATE tool (MD Anderson Cancer Center)
